# Supplementary material for: Anaplasma phagocytophilum and Other Anaplasma spp. in Various Hosts in the Mnisi Community, Mpumalanga Province, South Africa
Source: Microorganisms. 2020 Nov 18;8(11):1812. doi: 10.3390/microorganisms8111812 (PMC7698776; doi:10.3390/microorganisms8111812)
Supplement: Supplementary file 1 [file microorganisms-08-01812-s001.pdf]

Table S1: List of rodent species captured from the three habitat areas.

Sample ID	Collection Date	Sampling Locality	Morphological Identification
**R1**	2015/09/07	Tlhavekisa	*Mastomys* sp.
**R2**	2015/09/07	Tlhavekisa	*Mastomys coucha*
**R3**	2015/09/07	Tlhavekisa	*Mastomys* sp.
**R4**	2015/09/07	Tlhavekisa	*Mastomys* sp.
**R5**	2015/09/07	Tlhavekisa	*Mastomys coucha*
**R6**	2015/09/07	Tlhavekisa	*Mastomys coucha*
**R7**	2015/09/07	Tlhavekisa	*Gerbilliscus leucogaster*
**R8**	2015/09/07	Tlhavekisa	*Saccostomus campestris*
**R9**	2015/09/08	Tlhavekisa	*Mastomys* sp.
**R10**	2015/09/08	Tlhavekisa	*Mastomys* sp.
**R11**	2015/09/08	Tlhavekisa	*Mastomys coucha*
**R12**	2015/09/08	Tlhavekisa	*Mastomys coucha*
**R13**	2015/09/08	Manyeleti	*Gerbilliscus leucogaster*
**R14**	2015/09/08	Manyeleti	*Gerbilliscus leucogaster*
**R15**	2015/09/08	Manyeleti	*Gerbilliscus leucogaster*
**R16**	2015/09/08	Manyeleti	*Mastomys* sp.
**R17**	2015/09/09	Tlhavekisa	*Gerbilliscus leucogaster*
**R18**	2015/09/09	Gottenburg	*Mastomys* sp.
**R19**	2015/09/09	Gottenburg	*Mastomys natalensis*
**R20**	2015/09/09	Gottenburg	*Mastomys natalensis*
**R21**	2015/09/09	Manyeleti	*Mastomys coucha*
**R22**	2015/09/09	Manyeleti	*Saccostomus campestris*
**R23**	2015/09/09	Manyeleti	*Aethomys* sp.
**R24**	2015/09/09	Manyeleti	*Aethomys* sp.
**R25**	2015/09/09	Manyeleti	*Gerbilliscus leucogaster*
**R26**	2015/09/09	Manyeleti	*Mastomys* sp.
**R27**	2015/09/09	Manyeleti	*Aethomys* sp.
**R28**	2015/09/09	Manyeleti	*Gerbilliscus leucogaster*
**R29**	2015/09/10	Gottenburg	*Mastomys natalensis*
**R30**	2015/09/10	Gottenburg	*Mastomys natalensis*
**R31**	2015/09/10	Gottenburg	*Mastomys natalensis*
**R32**	2015/09/10	Gottenburg	*Lemniscomys rosalia*
**R33**	2015/09/10	Hlalakahle	*Saccostomus campestris*
**R34**	2015/09/10	Gottenburg	*Mastomys* sp.
**R35**	2015/09/10	Hlalakahle	*Saccostomus campestris*
**R36**	2015/09/10	Hlalakahle	*Gerbilliscus leucogaster*
**R37**	2015/09/10	Gottenburg	*Mastomys* sp.
**R38**	2015/09/10	Gottenburg	*Mastomys* sp.
**R39**	2015/09/10	Gottenburg	*Rattus* sp.
**R40**	2015/09/10	Gottenburg	*Mastomys* sp.

**R41**	2015/09/10	Gottenburg	*Lemniscomys rosalia*
**R42**	2015/09/10	Gottenburg	*Mastomys* sp.
**R43**	2015/09/10	Gottenburg	*Mastomys* sp.
**R44**	2015/09/10	Gottenburg	*Gerbilliscus leucogaster*
**R45**	2015/09/10	Gottenburg	*Mastomys* sp.
**R46**	2015/09/10	Gottenburg	*Mastomys* sp.
**R47**	2015/09/10	Gottenburg	*Mastomys* sp.
**R48**	2015/09/11	Manyeleti	*Saccostomus campestris*
**R49**	2015/09/11	Manyeleti	*Aethomys* sp.
**R50**	2015/09/11	Manyeleti	*Mastomys* sp.
**R51**	2015/09/11	Manyeleti	*Gerbilliscus leucogaster*
**R52**	2015/09/12	Manyeleti	*Saccostomus campestris*
**R53**	2015/09/12	Manyeleti	*Mastomys coucha*
**R54**	2015/09/12	Manyeleti	*Saccostomus campestris*
**R55**	2015/09/12	Manyeleti	*Saccostomus campestris*
**R56**	2015/09/12	Manyeleti	*Steatomys* sp.
**R57**	2015/09/12	Manyeleti	*Aethomys* sp.
**R58**	2015/09/12	Manyeleti	*Mus minutoides*
**R59**	2015/09/12	Manyeleti	*Gerbilliscus leucogaster*
**R60**	2015/09/12	Manyeleti	*Gerbilliscus leucogaster*
**R61**	2015/09/12	Manyeleti	*Mastomys coucha*
**R62**	2015/09/12	Manyeleti	*Steatomys* sp.
**R63**	2015/09/12	Hlalakahle	*Gerbilliscus leucogaster*
**R64**	2015/09/12	Hlalakahle	*Mus minutoides*
**R65**	2015/09/12	Hlalakahle	*Gerbilliscus leucogaster*
**R66**	2015/09/12	Hlalakahle	*Saccostomus campestris*
**R67**	2015/09/16	Gottenburg	*Mastomys* sp.
**R68**	2015/09/16	Gottenburg	*Mastomys* sp.
**R69**	2015/09/16	Gottenburg	*Aethomys* sp.
**R70**	2015/09/16	Gottenburg	*Mastomys* sp.
**R71**	2015/09/16	Hlalakahle	*Mastomys* sp.
**R72**	2015/09/16	Hlalakahle	*Mastomys* sp.
**R73**	2015/09/16	Hlalakahle	*Mastomys* sp.
**R74**	2015/09/16	Hlalakahle	*Mastomys natalensis*
**R75**	2015/09/16	Hlalakahle	*Mastomys natalensis*
**R76**	2015/09/16	Hlalakahle	*Aethomys* sp.
**R77**	2015/09/16	Hlalakahle	*Mastomys* sp.
**R78**	2015/09/16	Hlalakahle	*Mastomys natalensis*
**R79**	2015/09/16	Hlalakahle	*Mastomys* sp.
**R80**	2015/09/16	Hlalakahle	*Mastomys* sp.
**R81**	2015/09/16	Hlalakahle	*Mastomys* sp.
**R82**	2015/09/16	Hlalakahle	*Mastomys* sp.
**R83**	2015/09/14	Tlhavekisa	*Mastomys* sp.
**R84**	2015/09/14	Tlhavekisa	*Mastomys natalensis*

**R85**	2015/09/14	Tlhavekisa	*Rattus* sp.
**R86**	2015/09/14	Tlhavekisa	*Mastomys* sp.
**R87**	2015/09/16	Tlhavekisa	*Rattus* sp.
**R88**	2015/09/16	Tlhavekisa	*Mastomys* sp.
**R89**	2015/09/16	Tlhavekisa	*Rattus* sp.
**R90**	2015/09/16	Tlhavekisa	*Rattus* sp.
**R91**	2015/09/16	Gottenburg	*Mastomys* sp.
**R92**	2015/09/16	Gottenburg	*Mastomys* sp.
**R93**	2015/09/17	Tlhavekisa	*Rattus* sp.
**R94**	2015/09/17	Tlhavekisa	*Mastomys* sp.
**R95**	2015/09/17	Hlalakahle	*Mastomys natalensis*
**R96**	2015/09/17	Hlalakahle	*Mastomys* sp.
**R97**	2015/09/17	Hlalakahle	*Mastomys* sp.
**R98**	2015/09/17	Hlalakahle	*Mastomys natalensis*
**R99**	2015/09/17	Hlalakahle	*Mastomys natalensis*
**R100**	2015/09/17	Hlalakahle	*Mastomys* sp.
**R101**	2015/09/17	Hlalakahle	*Mastomys* sp.
**R102**	2015/04/01	Tlhavekisa	*Rattus tanezumi*
**R103**	2015/04/01	Tlhavekisa	*Mastomys natalensis*
**R104**	2015/01/04	Tlhavekisa	*Mastomys natalensis*
**R105**	2015/01/04	Tlhavekisa	*Mastomys natalensis*
**R106**	2015/01/04	Tlhavekisa	*Rattus rattus*
**R107**	2015/01/04	Tlhavekisa	*Rattus rattus*
**R108**	2015/01/04	Tlhavekisa	*Rattus rattus*
**R109**	2015/01/04	Tlhavekisa	*Rattus rattus*
**R110**	2015/01/04	Hlalakahle	*Rattus tanezumi*
**R111**	2015/01/04	Hlalakahle	*Mastomys* sp.
**R112**	2015/01/04	Hlalakahle	*Saccostomus campestris*
**R113**	2015/01/04	Hlalakahle	*Rattus tanezumi*
**R114**	2015/01/04	Tlhavekisa	*Mastomys* sp.
**R115**	2015/01/04	Tlhavekisa	*Mastomys* sp.
**R116**	2015/01/04	Hlalakahle	*Aethomys* sp.
**R117**	2015/01/04	Hlalakahle	*Gerbilliscus leucogaster*
**R118**	2015/01/04	Hlalakahle	*Saccostomus campestris*
**R119**	2015/01/04	Hlalakahle	*Mastomys* sp.
**R120**	2015/01/04	Hlalakahle	*Saccostomus campestris*
**R121**	2015/01/04	Hlalakahle	*Gerbilliscus leucogaster*
**R122**	2015/01/04	Hlalakahle	*Aethomys* sp.
**R123**	2015/01/04	Hlalakahle	*Saccostomus campestris*
**R124**	2015/01/04	Hlalakahle	*Saccostomus campestris*
**R125**	2015/01/04	Hlalakahle	*Gerbilliscus leucogaster*
**R126**	2015/01/04	Hlalakahle	*Gerbilliscus leucogaster*
**R127**	2015/01/04	Hlalakahle	*Saccostomus campestris*
**R128**	2015/01/04	Hlalakahle	*Gerbilliscus leucogaster*

**R129**	2015/01/04	Hlalakahle	*Gerbilliscus leucogaster*
**R130**	2015/01/04	Hlalakahle	*Gerbilliscus leucogaster*
**R131**	2015/01/04	Hlalakahle	*Saccostomus campestris*
**R132**	2015/01/04	Gottenburg	*Saccostomus campestris*
**R133**	2015/01/04	Gottenberg	*Saccostomus campestris*
**R134**	2015/01/04	Gottenberg	*Aethomys* sp.
**R135**	2015/01/04	Gottenberg	*Aethomys* sp.
**R136**	2015/01/04	Hlalakahle	*Gerbilliscus leucogaster*
**R137**	2015/01/04	Hlalakahle	*Saccostomus campestris*
**R138**	2015/01/04	Hlalakahle	*Gerbilliscus leucogaster*
**R139**	2015/01/04	Hlalakahle	*Gerbilliscus leucogaster*
**R140**	2015/01/04	Gottenburg	*Mastomys* sp.
**R141**	2015/01/04	Gottenburg	*Aethomys* sp.
**R142**	2015/01/04	Gottenburg	*Aethomys* sp.
**R143**	2015/01/04	Gottenburg	*Mastomys* sp.
**R144**	2015/01/04	Gottenburg	*Mastomys* sp.
**R145**	2015/01/04	Gottenburg	*Mastomys* sp.
**R146**	2015/01/04	Gottenburg	*Mastomys* sp.
**R147**	2015/01/04	Gottenburg	*Mastomys* sp.
**R148**	2015/01/04	Manyeleti	*Aethomys* sp.
**R149**	2015/01/04	Manyeleti	*Saccostomus campestris*
**R150**	2015/01/04	Manyeleti	*Gerbilliscus leucogaster*
**R151**	2015/01/04	Gottenburg	*Mastomys* sp.
**R152**	2015/01/04	Gottenburg	*Mastomys* sp.
**R153**	2015/01/04	Gottenburg	*Mastomys* sp.
**R154**	2015/01/04	Gottenburg	*Mastomys* sp.
**R155**	2015/01/04	Gottenburg	*Mastomys* sp.
**R156**	2015/01/04	Manyeleti	*Gerbilliscus leucogaster*
**R157**	2015/01/04	Manyeleti	*Saccostomus campestris*
**R158**	2015/01/04	Manyeleti	*Saccostomus campestris*
**R159**	2015/01/04	Manyeleti	*Mastomys coucha*
**R160**	2015/01/04	Gottenburg	*Mastomys* sp.
**R161**	2015/01/04	Gottenburg	*Mastomys* sp.
**R162**	2015/01/04	Gottenburg	*Mastomys* sp.
**R163**	2015/01/04	Gottenburg	*Mastomys* sp.
**R164**	2015/01/04	Gottenburg	*Mastomys* sp.
**R165**	2015/01/04	Gottenburg	*Mastomys* sp.
**R166**	2015/01/04	Gottenburg	*Mastomys* sp.
**R167**	2015/01/04	Gottenburg	*Mastomys* sp.
**R168**	2015/01/04	Gottenburg	*Rattus rattus*
**R169**	2015/01/04	Gottenburg	*Mastomys* sp.
**R170**	2015/01/04	Gottenburg	*Rattus rattus*
**R171**	2015/01/04	Gottenburg	*Mastomys natalensis*
**R172**	2015/01/04	Gottenburg	*Mastomys natalensis*

**R173**	2015/01/04	Gottenburg	*Mastomys* sp.
**R174**	2015/01/04	Gottenburg	*Rattus rattus*
**R175**	2015/01/04	Gottenburg	*Mastomys* sp.
**R176**	2015/01/04	Manyeleti	*Gerbilliscus leucogaster*
**R177**	2015/01/04	Manyeleti	*Mastomys coucha*
**R178**	2015/01/04	Manyeleti	*Gerbilliscus leucogaster*
**R179**	2015/01/04	Manyeleti	*Mastomys coucha*
**R180**	2015/01/04	Manyeleti	*Mastomys* sp.
**R181**	2015/01/04	Manyeleti	*Steatomys* sp.
**R182**	2015/01/04	Manyeleti	*Mastomys* sp.
**R183**	2015/01/04	Manyeleti	*Gerbilliscus leucogaster*
**R184**	2015/01/04	Manyeleti	*Gerbilliscus leucogaster*
**R185**	2014/07/29	Manyeleti	*Mastomys* sp.
**R186**	2014/07/29	Manyeleti	*Mastomys* sp.
**R187**	2014/07/29	Manyeleti	*Gerbilliscus leucogaster*
**R188**	2014/07/29	Manyeleti	*Mastomys* sp.
**R189**	2014/07/29	Manyeleti	*Mastomys* sp.
**R190**	2014/07/29	Manyeleti	*Mastomys* sp.
**R191**	2014/07/29	Manyeleti	*Gerbilliscus leucogaster*
**R192**	2014/07/29	Manyeleti	*Gerbilliscus leucogaster*
**R193**	2014/07/29	Manyeleti	*Mastomys* sp.
**R194**	2014/07/29	Manyeleti	*Mastomys* sp.
**R195**	2014/07/29	Manyeleti	*Mastomys* sp.
**R196**	2014/07/29	Manyeleti	*Mastomys* sp.
**R197**	2014/07/29	Manyeleti	*Mastomys* sp.
**R198**	2014/07/29	Manyeleti	*Gerbilliscus leucogaster*
**R199**	2014/07/29	Manyeleti	*Gerbilliscus leucogaster*
**R200**	2014/07/29	Manyeleti	*Gerbilliscus leucogaster*
**R201**	2014/07/31	Manyeleti	*Gerbilliscus leucogaster*
**R202**	2014/07/31	Manyeleti	*Gerbilliscus leucogaster*
**R203**	2014/07/31	Manyeleti	*Gerbilliscus leucogaster*
**R204**	2014/07/31	Manyeleti	*Gerbilliscus leucogaster*
**R205**	2014/07/30	Manyeleti	*Aethomys chrysophilus*
**R206**	2014/07/30	Manyeleti	*Aethomys chrysophilus*
**R207**	2014/07/30	Manyeleti	*Gerbilliscus leucogaster*
**R208**	2014/07/30	Manyeleti	*Aethomys chrysophilus*
**R209**	2014/07/30	Manyeleti	*Aethomys chrysophilus*
**R210**	2014/07/30	Manyeleti	*Gerbilliscus leucogaster*
**R211**	2014/07/30	Manyeleti	*Gerbilliscus leucogaster*
**R212**	2014/08/01	Manyeleti	*Lemniscomys rosalis*
**R213**	2014/08/01	Manyeleti	*Gerbilliscus leucogaster*
**R214**	2014/08/01	Manyeleti	*Gerbilliscus leucogaster*
**R215**	2014/08/02	Manyeleti	*Mus musculus*
**R216**	2014/08/02	Manyeleti	*Mus musculus*

**R217**	2014/07/29	Hluvukani	*Mus musculus*
**R218**	2014/07/29	Hluvukani	*Gerbilliscus leucogaster*
**R219**	2014/07/29	Hluvukani	*Gerbilliscus leucogaster*
**R220**	2014/07/29	Manyeleti	*Aethomys chrysophilus*
**R221**	2014/07/31	Manyeleti	*Aethomys chrysophilus*
**R222**	2014/07/31	Gottenburg	*Aethomys chrysophilus*
**R223**	2014/07/31	Gottenburg	*Mastomys* sp.
**R224**	2014/08/03	Tlhavekisa	*Mus musculus*
**R225**	2014/07/29	Manyeleti	*Mus musculus*
**R226**	2014/07/29	Hluvukani	*Aethomys chrysophilus*
**R227**	2014/07/29	Hluvukani	*Aethomys chrysophilus*
**R228**	2014/07/30	Gottenburg	*Rattus rattus*
**R229**	2014/07/30	Gottenburg	*Aethomys chrysophilus*
**R230**	2014/07/30	Gottenburg	*Aethomys chrysophilus*
**R231**	2014/07/30	Gottenburg	*Rattus rattus*
**R232**	2014/07/31	Gottenburg	*Aethomys chrysophilus*
**R233**	2014/07/31	Gottenburg	*Aethomys chrysophilus*
**R234**	2014/07/31	Gottenburg	*Aethomys chrysophilus*
**R235**	2014/07/31	Gottenburg	*Aethomys chrysophilus*
**R236**	2014/07/31	Gottenburg	*Aethomys chrysophilus*
**R237**	2014/07/31	Gottenburg	*Rattus rattus*
**R238**	2014/07/31	Gottenburg	*Aethomys chrysophilus*
**R239**	2014/07/31	Gottenburg	*Aethomys chrysophilus*
**R240**	2014/07/31	Gottenburg	*Aethomys chrysophilus*
**R241**	2014/07/31	Gottenburg	*Aethomys chrysophilus*
**R242**	2014/07/31	Gottenburg	*Aethomys chrysophilus*
**R243**	2014/07/30	Gottenburg	*Aethomys chrysophilus*
**R244**	2014/07/30	Gottenburg	*Aethomys chrysophilus*
**R245**	2014/07/30	Gottenburg	*Rattus rattus*
**R246**	2014/07/30	Gottenburg	*Aethomys chrysophilus*
**R247**	2014/07/30	Gottenburg	*Rattus rattus*
**R248**	2014/07/30	Gottenburg	*Aethomys chrysophilus*
**R249**	2014/08/01	Gottenburg	*Aethomys chrysophilus*
**R250**	2014/08/01	Gottenburg	*Aethomys chrysophilus*
**R251**	2014/08/01	Gottenburg	*Aethomys chrysophilus*
**R252**	2014/08/01	Gottenburg	*Aethomys chrysophilus*
**R253**	2014/08/01	Gottenburg	*Aethomys chrysophilus*
**R254**	2014/08/01	Gottenburg	*Aethomys chrysophilus*
**R255**	2014/08/01	Gottenburg	*Rattus rattus*
**R256**	2014/08/01	Gottenburg	*Aethomys chrysophilus*
**R257**	2014/08/01	Gottenburg	*Aethomys chrysophilus*
**R258**	2014/08/01	Gottenburg	*Aethomys chrysophilus*
**R259**	2014/08/02	Gottenburg	*Aethomys chrysophilus*
**R260**	2014/08/02	Gottenburg	*Aethomys chrysophilus*

**R261**	2014/08/02	Gottenburg	*Aethomys chrysophilus*
**R262**	2014/08/02	Gottenburg	*Rattus rattus*
**R263**	2014/08/03	Gottenburg	*Aethomys chrysophilus*
**R264**	2014/08/03	Gottenburg	*Aethomys chrysophilus*
**R265**	2014/08/03	Gottenburg	*Aethomys chrysophilus*
**R266**	2014/08/03	Gottenburg	*Aethomys chrysophilus*
**R267**	2014/08/03	Gottenburg	*Rattus rattus*
**R268**	2014/08/04	Gottenburg	*Rattus rattus*
**R269**	2014/08/04	Gottenburg	*Rattus rattus*
**R270**	2014/08/04	Gottenburg	*Aethomys chrysophilus*
**R271**	2014/08/06	Hlalakahle	*Aethomys chrysophilus*
**R272**	2014/08/06	Hlalakahle	*Aethomys chrysophilus*
**R273**	2014/08/06	Hlalakahle	*Aethomys chrysophilus*
**R274**	2014/08/06	Hlalakahle	*Aethomys chrysophilus*
**R275**	2014/08/06	Hlalakahle	*Aethomys chrysophilus*
**R276**	2014/08/06	Hlalakahle	*Aethomys chrysophilus*
**R277**	2014/08/06	Hlalakahle	*Rattus rattus*
**R278**	2014/08/06	Hlalakahle	*Aethomys chrysophilus*
**R279**	2014/08/07	Hlalakahle	*Aethomys chrysophilus*
**R280**	2014/08/07	Hlalakahle	*Aethomys chrysophilus*
**R281**	2014/08/07	Hlalakahle	*Aethomys chrysophilus*
**R282**	2014/08/07	Hlalakahle	*Aethomys chrysophilus*

Table S2: Origin and list of samples on which circular consensus sequencing (CCS) and multilocus gene sequencing were performed.

Sample ID	Origin	Host Species	*Msp2* gene qPCR Positive	Sequencing	
				CCS	Targeted
**C5**	Hlalakahle	Cattle	+	Yes	No
**C10**	Tlhavekisa	Cattle	+	Yes	No
**C13**	Seville A	Cattle	+	Yes	No
**C17**	Hlalakahle	Cattle	+	Yes	No
**C38**	Tlhavekisa	Cattle	+	Yes	No
**C42**	Seville B	Cattle	+	Yes	Yes
**C47**	Hlalakahle	Cattle	+	Yes	No
**C91**	Seville A	Cattle	+	Yes	No
**C96**	Gottenburg	Cattle	+	Yes	No
**D2**	Hluvukani	Dog	+	Yes	Yes
**D3**	Hluvukani	Dog	+	No	Yes
**D5**	Hluvukani	Dog	+	No	Yes
**D6**	Hluvukani	Dog	+	Yes	No
**D9**	Hluvukani	Dog	+	No	Yes
**D13**	Hluvukani	Dog	+	Yes	No
**D14**	Hluvukani	Dog	+	Yes	No
**D21**	Hluvukani	Dog	+	Yes	No
**D24**	Hluvukani	Dog	+	Yes	Yes
**D25**	Hluvukani	Dog	+	Yes	Yes
**D26**	Hluvukani	Dog	+	Yes	No
**D27**	Hluvukani	Dog	+	No	Yes
**D28**	Hluvukani	Dog	+	Yes	Yes
**D33**	Hluvukani	Dog	+	No	Yes
**D36**	Hluvukani	Dog	+	Yes	Yes
**D37**	Hluvukani	Dog	+	No	Yes
**H1**	Utha	Human	-	Yes	No
**H4**	Welverdiend	Human	-	Yes	No
**H8**	Gottenburg	Human	+	Yes	No
**H18**	Welverdiend	Human	+	Yes	No
**H27**	Welverdiend	Human	+	Yes	Yes
**H47**	Welverdiend	Human	+	Yes	Yes
**H53**	Utha	Human	+	Yes	Yes
**H59**	Utha	Human	+	Yes	Yes
**H69**	Utha	Human	+	Yes	No
**R2**	Tlhavekisa	*Mastomys coucha*	-	Yes	No
**R5**	Tlhavekisa	*Mastomys coucha*	-	Yes	No
**R6**	Tlhavekisa	*Mastomys coucha*	-	Yes	No
**R11**	Tlhavekisa	*Mastomys coucha*	-	Yes	No
**R12**	Tlhavekisa	*Mastomys coucha*	-	Yes	No
**R19**	Gottenburg	*Mastomys natalensis*	-	Yes	No
**R20**	Gottenburg	*Mastomys natalensis*	-	Yes	No
**R21**	Manyeleti	*Mastomys coucha*	-	Yes	No
**R29**	Gottenburg	*Mastomys natalensis*	-	Yes	No
**R30**	Gottenburg	*Mastomys natalensis*	-	Yes	No
**R31**	Gottenburg	*Mastomys natalensis*	-	Yes	No
**R53**	Manyeleti	*Mastomys coucha*	-	Yes	No
**R61**	Manyeleti	*Mastomys coucha*	-	Yes	No
**R74**	Hlalakahle	*Mastomys natalensis*	-	Yes	No
**R75**	Hlalakahle	*Mastomys natalensis*	+	Yes	No
**R78**	Hlalakahle	*Mastomys natalensis*	+	Yes	No
**R84**	Tlhavekisa	*Mastomys natalensis*	+	Yes	No
**R95**	Hlalakahle	*Mastomys natalensis*	+	Yes	No
**R98**	Hlalakahle	*Mastomys natalensis*	+	Yes	No
**R99**	Hlalakahle	*Mastomys natalensis*	+	Yes	No
**R102**	Tlhavekisa	*Rattus tanezumi*	+	No	Yes

**R103**	Tlhavekisa	*Mastomys natalensis*	+	No	Yes
**R104**	Tlhavekisa	*Mastomys natalensis*	+	No	Yes
**R105**	Tlhavekisa	*Mastomys natalensis*	+	No	Yes
**R124**	Hlalakahle	*Saccostomus campestris*	+	No	Yes
**R125**	Hlalakahle	*Gerbilliscus leucogaster*	+	No	Yes
**R138**	Hlalakahle	*Gerbilliscus leucogaster*	+	No	Yes
**R159**	Manyeleti	*Mastomys coucha*	+	Yes	No
**R171**	Gottenburg	*Mastomys natalensis*	+	Yes	No
**R172**	Gottenburg	*Mastomys natalensis*	+	Yes	No
**R177**	Manyeleti	*Mastomys coucha*	+	Yes	No
**R179**	Manyeleti	*Mastomys coucha*	+	Yes	No
**RA1**	Athol	*Rhipicephalus sanguineus*	+	No	Yes
**RA3**	Athol	*Rhipicephalus sanguineus*	+	No	Yes
**RH1**	Hluvukani	*Rhipicephalus sanguineus*	+	No	Yes
**RH3**	Hluvukani	*Rhipicephalus sanguineus*	+	No	Yes
**RH8**	Hluvukani	*Rhipicephalus sanguineus*	+	No	Yes

+ Positive on qPCR assay

- Negative on qPCR assay

Table S3: Genbank accession numbers of sequences used in the phylogenetic analysis of the *Anaplasma* species.

Accession number	Organism	Strain/	Host
16S rRNA			
U02521	*A. phagocytophilum*	Webster/US	Human
NC007797	*A. phagocytophilum*	HZ/US	Human
CP006618	*A. phagocytophilum*	Dog2/US	Dog
AF093789	HGE agent	US	Human
CP046391	*A. platys* strain S3	Saint Kitts	Dog
M82801	*A. platys*	US	Dog
KT982643	*A. platys*	India	Dog
LC269822	*A. platys*	Zambia	Dog
AY570540	*Anaplasma* sp. South Africa dog 1245	South Africa	Dog
AY570539	*Anaplasma* sp. South Africa dog 1076	South Africa	Dog
LC269823	*Anaplasma* sp. ZAM dog	Zambia	Dog
MT918373	*Anaplasma* sp. Southern Africa dog	South Africa	Black-backed jackal
MT918374	*Anaplasma* sp. Southern Africa dog	South Africa	Black-backed jackal
KU586025	*Candidatus* Anaplasma boleense	WHANSP/China	*Anopheles sinensis*
KU586169	*Candidatus* Anaplasma boleense	WHANSA China	*Anopheles sinensis*
MF576175	*Anaplasma* sp. Mymensingh	Bangladesh	Cattle
KY924886	Uncultured *Anaplasma* sp. Dedessa	Ethiopia	Cattle
KJ410249	*Anaplasma* sp. BL102-7	China	*Hyalomma asiaticum*
KP006405	Uncultured *Anaplasma* sp. Dog9	Philippines	Dog
U03775	*A. bovis*	South Africa	Cattle
AF309869	*A. centrale*	Strain Isreal	Cattle
AY048816	*A. marginale*	St. Maries	Cattle
AF414870	*A. ovis*	South Africa	Sheep
U54806	*Anaplasma* sp. Omatjenne	Namibia	*Hyalomma truncatum*
KP314237	*A. capra*	China	Tick
U11021	*Rickettsia rickettsii*	Sawtooth/US	*Dermacentor andersoni*
gltA gene			
AF304140	*A. marginale*	Florida/US	Cattle
KJ410283	*A. ovis*	China	Tick
CP001759	*A. centrale*	Isreal	Cattle
KU586317	*A. bovis*	China	*Aedes albopictus*
AQW38804	*A. bovis*	China	*Aedes albopictus*
AF304137	*A. phagocytophilum*	California/US	Horse
CP015376	*A. phagocytophilum*	Norway variant2/ Norway	Sheep
AGR81592	*A. phagocytophilum*	Dog2/US	Dog
SCV65315	*A. phagocytophilum*	France	Genome Assembly
AKZ20811	*A. phagocytophilum*	Spain	*Ixodes* sp.
KX987359	*Candidatus* Anaplasma boleense	WHBMXZ/China	*Rhipicephalus microplus*
KR261628	*A. capra*	China	*Haemaphysalis longicornis*
KP861638	*A. phagocytophilum*	NY18/US	*Ixodes* sp.
KP861637	*A. phagocytophilum*	DogL610/Germany	*Ixodes* sp.
CP046391	*A. platys* strain S3	Saint Kitts	Dog
DQ525687	*A. platys*	Sicily/Italy	Dog
LC269826	*A. platys*	ZAM dog/Zambia	Dog
AY570541	*Anaplasma* sp. SA dog	South Africa	Dog
AAT74599	*Anaplasma* sp. SA dog	South Africa	Dog
LC269827	*Anaplasma* sp. ZAM dog	Zambia	Dog
U59729	*Rickettsia rickettsii*	Iowa/US	Vero cells

Table S4: Sample information for the 16S rRNA, *gltA*, *msp4* and *ankA* sequences generated in this study.

Accession number	Sample ID	Species	Origin	Host	Sequence type
**16S rRNA**					
**MK814405**	D2-1	*A. phagocytophilum*	Hluvukani	Dog	Aph1/16S
**MK814406**	D2-2	*A. phagocytophilum*	Hluvukani	Dog	Aph1/16S
**MK814404**	D2-4	*A. phagocytophilum*	Hluvukani	Dog	Aph1/16S
**MK814402**	D2-5	*A. phagocytophilum*	Hluvukani	Dog	Aph1/16S
**MK814403**	D2-8	*A. phagocytophilum*	Hluvukani	Dog	Aph1/16S
**MK814410**	*D24	*A. phagocytophilum*	Hluvukani	Dog	Aph2/16S
**MK814412**	*D28	*A. phagocytophilum*	Hluvukani	Dog	Aph2/16S
**MK814407**	H59	*A. phagocytophilum*	Hluvukani	Human	Aph/1or2/16S
**MK814411**	*R98	*A. phagocytophilum*	Hlalakahle	*Mastomys natalensis*	Aph2/16S
**MK814408**	R102	*A. phagocytophilum*	Thlavekisa	*Rattus tanezumi*	Aph/1or2/16S
**MK814409**	R103	*A. phagocytophilum*	Thlavekisa	*Mastomys natalensis*	Aph/1or2/16S
**MK814426**	D3-1	*Anaplasma* sp. SA dog	Hluvukani	Dog	Adog1/16S
**MK814428**	D3-5	*Anaplasma* sp. SA dog	Hluvukani	Dog	Adog1/16S
**MK814425**	D3-6	*Anaplasma* sp. SA dog	Hluvukani	Dog	Adog1/16S
**MK814423**	D3-7	*Anaplasma* sp. SA dog	Hluvukani	Dog	Adog16S1
**MK814427**	D5-1	*Anaplasma* sp. SA dog	Hluvukani	Dog	Adog1/16S
**MK814441**	D9-6	*Anaplasma* sp. SA dog	Hluvukani	Dog	Adog2/16S
**MK814431**	D27-2	*Anaplasma* sp. SA dog	Hluvukani	Dog	Adog1/16S
**MK814435**	D27-9	*Anaplasma* sp. SA dog	Hluvukani	Dog	Adog2/16S
**MK814422**	D27-1	*Anaplasma* sp. SA dog	Hluvukani	Dog	Adog1/16S
**MK814429**	D27-13	*Anaplasma* sp. SA dog	Hluvukani	Dog	Adog1/16S
**MK814433**	D36-3	*Anaplasma* sp. SA dog	Hluvukani	Dog	Adog1/16S
**MK814432**	D36-9	*Anaplasma* sp. SA dog	Hluvukani	Dog	Adog1/16S
**MK814430**	D36-10	*Anaplasma* sp. SA dog	Hluvukani	Dog	Adog1/16S
**MK814434**	D36-11	*Anaplasma* sp. SA dog	Hluvukani	Dog	Adog1/16S
**MK814446**	D37-1	*Anaplasma* sp. SA dog	Hluvukani	Dog	Adog1/16S
**MK814447**	D37-4	*Anaplasma* sp. SA dog	Hluvukani	Dog	Adog1/16S
**MK814424**	D37-8	*Anaplasma* sp. SA dog	Hluvukani	Dog	Adog1/16S
**MK814445**	D37-9	*Anaplasma* sp. SA dog	Hluvukani	Dog	Adog1/16S
**MK814436**	D3	*Anaplasma* sp. SA dog	Hluvukani	Dog	Adog2/16S
**MK814438**	D5	*Anaplasma* sp. SA dog	Hluvukani	Dog	Adog2/16S
**MK814443**	D27	*Anaplasma* sp. SA dog	Hluvukani	Dog	Adog2/16S
**MK814439**	*D36	*Anaplasma* sp. SA dog	Hluvukani	Dog	Adog2/16S
**MK814437**	D37	*Anaplasma* sp. SA dog	Hluvukani	Dog	Adog2/16S
**MK814442**	RA3	*Anaplasma* sp. SA dog	Athol	*Rhipicephalus sanguineus*	Adog2/16S
**MK814440**	RH3	*Anaplasma* sp. SA dog	Hluvukani	*Rhipicephalus sanguineus*	Adog2/16S
**MK814444**	RH8	*Anaplasma* sp. SA dog	Hluvukani	*Rhipicephalus sanguineus*	Adog2/16S
**MK814416**	D25-2	*A. platys*	Hluvukani	Dog	Apla1/16S
**MK814419**	D25-5	*A. platys*	Hluvukani	Dog	Apla1/16S
**MK814414**	D25-6	*A. platys*	Hluvukani	Dog	Apla1/16S
**MK814417**	D25-8	*A. platys*	Hluvukani	Dog	Apla1/16S
**MK814418**	D25-9	*A. platys*	Hluvukani	Dog	Apla1/16S
**MK814415**	D33-3	*A. platys*	Hluvukani	Dog	Apla1/16S
**MK814421**	D33-4	*A. platys*	Hluvukani	Dog	Apla1/16S
**MK814420**	D33-5	*A. platys*	Hluvukani	Dog	Apla1/16S
**MK814413**	D33-7	*A. platys*	Hluvukani	Dog	Apla1/16S
**MK814448**	*C5	*Anaplasma* sp. Mymensingh	Hlalahkahle	Cattle	Asm1/16S
**MK814450**	*C13	*Candidatus* Anaplasma boleense	Seville A	Cattle	Cab1/16S
**MK814449**	*C91	*Anaplasma* sp. Mymensingh	Seville A	Cattle	Asm1/16S

**GltA**					
**MK804077**	D24	*A. phagocytophilum*	Hluvukani	Dog	Aph1/GltA
**MK804079**	D25	*A. phagocytophilum*	Hluvukani	Dog	Aph1/GltA
**MK804078**	D28	*A. phagocytophilum*	Hluvukani	Dog	Aph1/GltA
**MK804080**	R102	*A. phagocytophilum*	Thlavekisa	*Rattus tanezumi*	Aph1/GltA
**MK804081**	D36	*Anaplasma* sp. SA dog	Hluvukani	Dog	Adog1/GltA
**Msp4**					
**MK804088**	D3	*Anaplasma* sp.	Hluvukani	Dog	AspM/Msp4
**MK804089**	D33	*Anaplasma* sp.	Hluvukani	Dog	AspM/Msp4
**MK804096**	R102	*Anaplasma* sp.	Thlavekisa	*Rattus tanezumi*	AspM/Msp4
**MK804097**	R102-8	*Anaplasma* sp.	Thlavekisa	*Rattus tanezumi*	AspM/Msp4
**MK804099**	R102-10	*Anaplasma* sp.	Thlavekisa	*Rattus tanezumi*	AspM/Msp4
**MK804093**	R103	*Anaplasma* sp.	Thlavekisa	*Mastomys natalensis*	AspM/Msp4
**MK804098**	R103-8	*Anaplasma* sp.	Thlavekisa	*Mastomys natalensis*	AspM/Msp4
**MK804103**	R104-7	*Anaplasma* sp.	Thlavekisa	*Mastomys natalensis*	AspM/Msp4
**MK804092**	R105	*Anaplasma* sp.	Thlavekisa	*Mastomys natalensis*	AspM/Msp4
**MK804102**	R105-4	*Anaplasma* sp.	Thlavekisa	*Mastomys natalensis*	AspM/Msp4
**MK804100**	R105-5	*Anaplasma* sp.	Thlavekisa	*Mastomys natalensis*	AspM/Msp4
**MK804083**	R124	*Anaplasma* sp.	Hlalakahle	*Saccostomus* sp.	AspM/Msp4
**MK804101**	R125-2	*Anaplasma* sp.	Hlalakahle	*Gerbillicus leucogaster*	AspM/Msp4
**MK804087**	R138	*Anaplasma* sp.	Hlalakahle	*Gerbillicus leucogaster*	AspM/Msp4
**MK804094**	H27	*Anaplasma* sp.	Hluvukani	Human	AspM/Msp4
**MK804104**	H47	*Anaplasma* sp.	Hluvukani	Human	AspM/Msp4
**MK804085**	H53	*Anaplasma* sp.	Hluvukani	Human	AspM/Msp4
**MK804086**	RA1	*Anaplasma* sp.	Athol	*Rhipicephalus sanguineus*	AspM/Msp4
**MK804095**	RH1	*Anaplasma* sp.	Hluvukani	*Rhipicephalus sanguineus*	AspM/Msp4
**MK804082**	RH3	*Anaplasma* sp.	Hluvukani	*Rhipicephalus sanguineus*	AspM/Msp4
**MK804091**	RA3	*Anaplasma* sp.	Athol	*Rhipicephalus sanguineus*	AspM/Msp4
**MK804084**	RH8	*Anaplasma* sp.	Hluvukani	*Rhipicephalus sanguineus*	AspM/Msp4
**MK804090**	C42	*Anaplasma* sp.	Seville B	Cattle	AspM/Msp4
**AnkA**					
**MK804106**	D3	*Anaplasma* sp.	Hluvukani	Dog	AspA/AnkA
**MK804109**	D33	*Anaplasma* sp.	Hluvukani	Dog	AspA/AnkA
**MK804108**	D36	*Anaplasma* sp.	Hluvukani	Dog	AspA/AnkA
**MK804105**	R102	*Anaplasma* sp.	Thlavekisa	*Rattus tanezumi*	AspA/AnkA
**MK804110**	R103	*Anaplasma* sp.	Thlavekisa	*Mastomys natalensis*	AspA/AnkA
**MK804107**	R124	*Anaplasma* sp.	Hlalakahle	*Saccostomus* sp.	AspA/AnkA
**MK804111**	RH1	*Anaplasma* sp.	Hluvukani	*Rhipicephalus sanguineus*	AspA/AnkA

*Sequences were extracted from data generated from the characterization of the bacterial blood microbiome of dogs, cattle and rodents in the Mnisi community area, Mpumalanga Province South Africa.

msp2

```
                           1                                                                              80
A. platys            (1) ATGA----AGGAAAGAAAACTTGCGCTAAGTGGAGCGGTGGCGATGACAGTTTTGGTGTCGACTGCTGGTACCGGGACTG
A. phagocytophilum   (1) ATGAGAAAAGGAAAGATAATCTTAGG-AAGCGTAATGATGTCGATGGCTATAGTCATGGCTGGGAATGATGTCAGGGCT-
                           81                                                                            160
A. platys           (77) CGGCAGGGTCGGACGTGGACTATGTAAGTAAGTTCGGTGAGGGCAGCTTCTACGTAGGTCTAAACTATAGTCCGGCGTTT
A. phagocytophilum  (79) ---CATGAT--GACGTTAGCGCTTTGGAGACTGGTGGTGCGGGATATTTCTATATTGGTTTGGATTACAGTCCAGCGTTT
                           161                                                                           240
A. platys          (157) AGTAAGATAAATGGGTTTGAGATAAGAGAGAGTACCGGGGAAACTGCGGCAGTATATCCGTACATGAAAGATGGAACTAG
A. phagocytophilum (154) AGCAAGATAAGAGATTTTAGTATAAGGGAGAGTAACGGAGAGACTAAGGCAGTATATCCATACTTAAAGGATGGAAAGAG
                           241                                                                           320
A. platys          (237) AGTGGAGTGGAAAGCTGAGAAGTTCGACTGGAACACACCAGATCCGAGGATTAAGTTTAAAAACAATCCTATCGTGGCGT
A. phagocytophilum (234) TGTAAAGCTAGAGTCACACAAGTTTGACTGGAACACTCCTGATCCTCGGATTGGGTTTAAGGACAACATGCTTGTAGCTA
                           321                                                                           400
A. platys          (317) TAGAAGGAAGTGTGGGCTACAGTATCGGGGTAGCGAGAGTAGAACTGGAGATCGGCTATGAACAGTTCAAGACGAAAGGA
A. phagocytophilum (314) TGGAAGGCAGTGTTGGTTATGGTATTGGTGGTGCCAGGGTTGAGCTTGAGATTGGTTACGAGCGCTTCAAAACCAAGGGT
                           401                                                                           480
A. platys          (397) ATAAGAGATACGGGAAGTAAGGAAGAAGAAGCTGATGCCGTGTACCTGTTGGCTAAGAAGCTACCGCATACCCTGGTGAG
A. phagocytophilum (394) ATTAGAGATAGTGGTAGTAAGGAAGATGAAGCTGATACAGTATATCTACTAGCTAAGGAGTTAGCTTATGATGTTGTTAC
                           481                                                                           560
A. platys          (477) TGACCAGAGCGATAAATTCCTGGAGGAGCTGAAGAATACGAAAGCGGCGGAGATCGTTAAATTTGCTGAGGCTGTTGGCA
A. phagocytophilum (474) TGGACAGACTGATAAGCTTGCTGCTGCTCTTGCTAAAACCTCTGGTAAAGATATCGTTCAGTTTGCCAATGCTGTGAAAA
                           561                                                                           640
A. platys          (557) CATCGGCAAAGGATATTGATGGAAAGGTTTGTAAGAAGGGCGGCAGCGGCAATGCCGCGGGC-AGCTGGAAGT-GTA---
A. phagocytophilum (554) TTTCCAGCCCTGAAATTGATGGGAAGGTTTGTA-GTGGGGATCATGCTGCTATAACTAAGGCTAAGGGGAAGAAGTATGT
                           641                                                                           720
A. platys          (632) -----------CGCAGACTGGCAGCAACG--GCGTCAGCACCGC--AGAGTTCAGTAAAATATTTACGAAGGC-------
A. phagocytophilum (633) TGCGGAGTTGTCGACGACACACAGCGACGAGGAGACAACACAGTGTAGCGGTTTGGGAAATACG-ACGGGGGCGACAGGA
                           721                                                                           800
A. platys          (690) ----AGACGTA-------------AATACTGACAACA------AAGGCAAAGCACGGCCTA----ACG--GGAACAACGA
A. phagocytophilum (712) CCAAAGACGTTGAGTGGTTTTGTTAATACTGTGAAAGTCGGTGAAGGTAAAAACTGGCCTAGGGGACGAGGGAGTAATGG
                           801                                                                           880
A. platys          (741) C-GCTGCGAAAGCG------GAAGACCTAA--GTATTGCGT------TGAATAGAGAACTA---------ACCAGCGCCG
A. phagocytophilum (792) CAGCAGTCAAAACGTCGAGGGAGAACCTAATAGTAATGCAAACGCTATGGCTAAAGACCTATTAGGTCTTAATCGTGATG
                           881                                                                           960
A. platys          (797) AAAAGAACAAGGTAGCTGGCCTACTAACCAGGACTATATCCGGTGGTGAGGTAGTGGAGATCCGTGCGGTGTCGACAACG
A. phagocytophilum (872) AAAAAACCATAGTAGCAGGATTACTTGCTAAAACTATTGAAGGGGGAGAGGTTGTTGAGATCAGAGCTGTTTCTTCTACA
                           961                                                                          1040
A. platys          (877) TCAGTAATGTTGAATGGTTGTTATGATCTGCAGAGTGAAGGGTTTAGTATAGTACCTTATGCATGTCTTGGTGTAGGTGC
A. phagocytophilum (952) TCTGTAATGGTTAATGCTTGTTATGATCTTCTTAGTGAAGGTTTAGGTGTTGTTCCTTATGCTTGTGTTGGTTTAGGTGG
                           1041                                                                         1120
A. platys          (957) TAACTTCGTTGGCATTGTTGACGGACACGTCACTCCTAAACTGGCTTACAAGGTCAAGGCTGGTTTGAGTTATGAATTGT
A. phagocytophilum(1032) TAACTTCGTAGGTGTTGTTGATGGGCATATCACTCCTAAGCTTGCTTATAGGTTAAAGGCTGGCTTGAGTTATCAGCTCT
                           1121                                                                         1200
A. platys         (1037) CGCCGGAAATCTCAATGTTCGCTGGTGGGTTCTATCATCGGGTGCTGGGTGAAGGTGAGTACGATGATCTGCCAGTGCAG
A. phagocytophilum(1112) CTCCTGAAATCTCCGCTTTTGCGGGTGGATTCTACCATCGTGTTGTGGGAGATGGCGTTTATGATGATCTGCCGGCTCAA
                           1201                                                                         1280
A. platys         (1117) AGGCTTGTAGACGATGCGACTACGAACAAG---ACTAAAGAGTTCGCTAAAGCGTCGTTCAAGATGGCGTACACTGGTGC
A. phagocytophilum(1192) CGTCTTGTAGATGATACTAGTCCGGCGGGTCGTACTAAGGATACTGCTATTGCTAACTTCTCCATGGCTTATGTCGGTGG
                           1281                      1308
A. platys         (1194) TGAAATCGGTGTTAGGTTTGCGTTCTAA
A. phagocytophilum(1272) GGAATTTGGTGTTAGGTTCGCTTTTTAA
```

Figure S1: Alignment of *msp2* sequences from *A. platys* and *A. phagocytophilum*. The *A. platys* sequence corresponds to accession # GU357491, while the *A. phagocytophilum* sequence was taken from the expression site of the HGE1 strain genome (Accession # APHH00000000). The underlined regions show the primers used in the study. The reverse complement (rc) of the reverse primer is underlined. The TaqMan probe is double underlined. The numbers above each row indicate the alignment position number. The numbers at the start of each row in parentheses indicate the base number of the first base on that row for that sequence. Only two sequences are shown because the primers do not sit in the variable region of this gene.

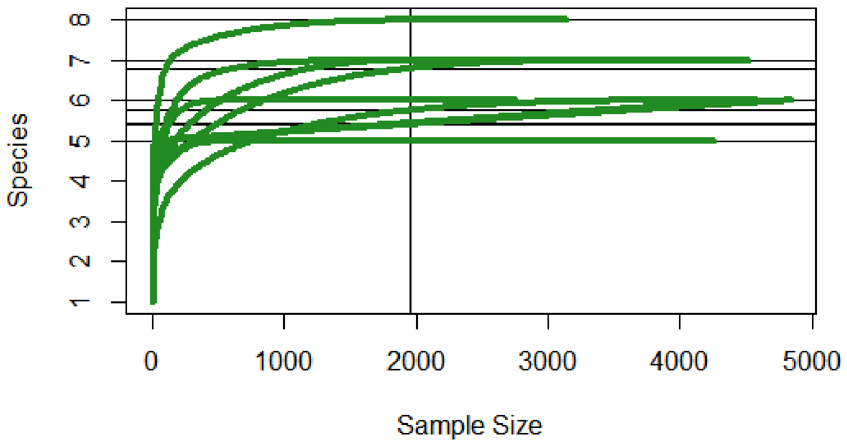

Figure S2: Representative rarefaction curves from samples tested. The mean species diversity of bacterial populations was plotted as a function of read depth. The vertical line in the plot indicates the value where rarefaction criterion was satisfied.

msp4

```
                1                                                                              80
AmStM     (1) ATGAATTACAGAGAATTGTTTACAGGGGGCCTGTCAGCAGCCACAGTCTGCGCCTGCTCCCTACTTGTTAGTGGGGCCGT
AcIs      (1) ATGAATTACAGAGAATTGTTTACAGGGGGCCTGTCAGCAGCCACAGTCTGCGCTTGCTCCCTACTTGTCAGTGGGCCTGT
AoHa      (1) ATGAATTACAGAGAATTGTTTACAGGGGGCCTGTCAGCAGCCACAGTCTGCGCCTGCTCCCTACTTGTTAGTGGGGCCGT
Apl       (1) ATGAATTACAGAGAATTACTTGTAGGGAGCTTGTCTGCTATGGCAGTCTGCGCATGCTCTCTTACAGTTAGTGAATCCTC
ApHGE1    (1) ATGAATTACAGAGAATTGCTTGTAGGGAGCCTATCTGCGGCCGCAGTATGTGCCTGCTCCCTTTTAATTAGTGGATCCTC
ApHZ      (1) ATGAATTACAGAGAATTGCTTGTAGGGAGCCTATCTGCGGCCGCAGTATGTGCCTGCTCCCTTTTAATTAGTGGATCCTC
                81                                                                            160
AmStM    (81) AGTGGCATCTCCCATGAGTCACGAAGTGGCTTCTGAAGGG---GGAGTAATGGGAGGTAGCTTTTACGTGGGTGCGGCCT
AcIs     (81) AGTAGCGTCTCCCATGGGGCATGAATCTGTGTCCGAAGGGAGCGGAGTTATGGGTGGTAGCTTTTATGTGAGCACAGCTT
AoHa     (81) AGTGGCGTCTCCCATGAGTCATGAAGTGGCTTCTGAAGGGAGCGGGGTCATGGGAGGTAGCTTTTATGTGAGTGCGGCTT
Apl      (81) ATTGGCGTCGGCAGCAAATCATGATGCTACATCTGATGTAGGTGGTGTTATGAACGGAAGTTTTTACGTTAGTGGTAACT
ApHGE1   (81) ATTTGCGTATTCAGGCAATAATGATGCG---TCTGATGTTAGCGGTGTTATGAACGGCAGCTTTTACGTAAGTGGTAGCT
ApHZ     (81) ATTTGCGTATTCAGGCAATAATGATGCG---TCTGATGTTAGCGGTGTTATGAACGGCAGCTTTTACGTAAGTGGTAGCT
                161                       AB1692F                                             240
AmStM   (158) ACAGCCCAGCATTTCCTTCTGTTACCTCGTTCGACATGCGTGAGTCAAGCAAAGAGACCTCATACGTTAGAGGCTATGAC
AcIs    (161) ACAGCCCAGCGTTTCCCTCTGTTACCTCGTTCGACATACGCGAGTCAGGTAAAGAGACTTCCTACGTCAAGGGCTACGAC
AoHa    (161) ACAGCCCAGCGTTTCCCTCTGTTACCTCATTCGACATGCGTGAGTCAAGCAGAGAGACCTCGTATGTTAGAGGCTATGAC
Apl     (161) ACAGTCCAACATTTCCTTCCATTACTTCTTTTGACATAAGGGAATCTGGTCGTGATAGTTCTAGCGTTAAAAGCTACAGC
ApHGE1  (158) ACAGTCCGTCATTTCCTTCTATATCTTCATTTGCTATTAGTGAGTCTGACAGGGGAGGATCTTACGTAAAAGGTTATAAC
ApHZ    (158) ACAGTCCGTCATTTCCTTCTATATCTTCATTTGCTATTAGTGAGTCTGACAGGGGAGGATCTTACGTAAAAGGTTATAAC
                241                                                                           320
AmStM   (238) AAGAGCCTTGCAACGATTGATGTGAGTGTGCCGGCAAACTTTTCCAAATCTGGCTACACTTTTGCCTTCTCTAAGAACTT
AcIs    (241) AAGAGCATCGCAACAATTGATGTGAGTGCGCATGAAAACTTTTCTAGATCTAGCTACTCCTTTGCGTTCTCTAAAAACTT
AoHa    (241) AAGAGTGTTGCAACAATTGATGTGAGTGCGCCAGCAAACTTTTCCAAATCCGGCTACACTTTTGCTTTCTCTAAGAATTT
Apl     (241) AAAACCGCTCCTACACTCAATATCTCGCAACCTGAAAACTTCAATCAAGAGGGACACACTTTTAAATTTGCAAAGAACTT
ApHGE1  (238) AAGAATTTGAGCACGTTGAATGTTTCAGATCCTGCCAGCTTCACGCAACATGATCCCTCATTCAAGTTCGCTAAGAGTTT
ApHZ    (238) AAGAATTTGAGCACGTTGAATGTTTCAGATCCTGCCAGCTTCACGCAACATGATCCCTCATTCAAGTTCGCTAAGAGTTT
                321                                                                           400
AmStM   (318) AATCACATCTTTCGACGGCGCTGTGGGATATTCTCTAGGAGGAGCCAGAGTGGAATTGGAAGCGAGCTACAGAAGGTTTG
AcIs    (321) ACTTACGTCTTTTGATGGTGCTGTGGGATACTCCCTAGGGGGGGCCAGAGTAGAGCTGGAAGCGAGCTACAGAAGATTTG
AoHa    (321) ACTCACATCTTTCGACGGCGCTGTGGGATATTCTCTGGGAGGAGCTAGAGTGGAACTAGAAGCAAGCTACAGAAGGTTTG
Apl     (321) GCTAACATCCTTTGAAGGAGCTGCAGGTTATGCAATGGGCGGTGCTAGAGTAGAAGTGGAAGCTGGATACAAAAAATTTG
ApHGE1  (318) ACTAACATCTTTTGACGGTGCAACTGGCTATGCTATTGGCGGAGCTAGAGTGGAAGTTGAAGTTGGATATAAAAAGTTTG
ApHZ    (318) ACTAACATCTTTTGACGGTGCAACTGGCTATGCTATTGGCGGAGCTAGAGTGGAAGTTGAAGTTGGATATAAAAAGTTTG
                401                                                                           480
AmStM   (398) CTACTTTGGCGGACGGGCAGTACGCGAAAAGTGGTGCGGAATCTCTGGCAGCTATTACCCGCGACGCTAACATTACTGAG
AcIs    (401) CCACCTTGGCGGACGGACAGTATGCGAAAAGTGGTGCGGAAGCTTTGGCTGCCGTTAGCCGCGAAGCTGCGCTCACTGCA
AoHa    (401) CTACTTTAGCGGACGGGCAGTACGCAAAAAGTGGTGCAGAGTCCCTGGCTGCAATTACTCGCGACGCTGCCATTACTGAG
Apl     (401) CTGCTACTTCTGATGTGGATTACAAACATGCAGATGCTCATCAGTTCGTCGGTATAGGACGTGAACCAGTATTCGCAGAC
ApHGE1  (398) AAACGCTCGCTGAAAGTGACTACAAGCATGTAGAATCACATAATTTTGTTGCTGTGGGTCGTGACGCGACATTAACTCCA
ApHZ    (398) AAACGCTCGCTGAAAGTGACTACAAGCATGTAGAATCACATAATTTTGTTGCTGTGGGTCGTGACGCGACATTAACTCCA
                481                                                                           560
AmStM   (478) ACCAATTACTTCGTAGTCAAAATTGATGAAATCACAAACACCTCAGTCATGTTAAATGGCTGCTATGACGTGCTGCACAC
AcIs    (481) ACCAATTACTTTGTGGTCAAGGTGGACGAAATTATAAACACTTCAGTCATGCTAAATGGTTGTTATGACGTGTTGCACAC
AoHa    (481) AACAATTACTTTGTGGTCAAAATCGATGAAATCACAAACACTTCAGTCATGCTAAATGGCTGCTATGACGTGTTGCACAC
Apl     (481) GGAAACTACTTCGTAATGAAGATCGATAGTATCACTGACATTTCTGTGATGCTCAATGCTTGTTATGACGTAATGCACAC
ApHGE1  (478) GACAACTTCTTTGTAATGAAAATAGACAGCGTCAAAGATATATCTGTAATGCTTAACGCTTGTTACGACGTTATGCATAC
ApHZ    (478) GACAACTTCTTTGTAATGAAAATAGACAGCGTCAAAGATATATCTGTAATGCTTAACGCTTGTTACGACGTTATGCATAC
                561                                                                           640
AmStM   (558) AGATTTACCTGTGTCCCCGTATGTATGTGCCGGGATAGGCGCAAGCTTTGTTGACATCTCTAAGCAAGTAACCACAAAGC
AcIs    (561) GGATTTGCCTGTGTCTCCGTACGTGTGCGCTGGGATAGGTGCAAGCTTTGTTGACATATCTAAGCAAGTAACCGCAAAAC
AoHa    (561) AGATTTGCCTGTGTCCCCATATGTGTGTGCCGGAATAGGTGCTAGCTTTGTCGACATTTCTAAGCAAGTAACCACAAAGC
Apl     (561) TGATTTGCCCGTGTCTCCTTACGTGTGTGCGGGTTGGGTGCAAGTTTTGTTGACATTGCAAACCAAACTACAAGTAAGT
ApHGE1  (558) TGACTTGCCTGTATCTCCTTACATGTGTGCTGGGTTAGGGGCGAGCTTTATTAATATCGCCGATCACGTTACAAGTAAGT
ApHZ    (558) TGACTTGCCTGTATCTCCTTACATGTGTGCTGGGTTAGGGGCGAGCTTTATTAATATCGCCGATCACGTTACAAGTAAGT
                641                                                                           720
AmStM   (638) TGGCCTACAGGGGCAAGGTTGGATTAGCTACCAGTTTACTCCGGAAATATCCTTGGTGGCAGGTGGGTTCTACCACGGG
AcIs    (641) TAGCCTACAGAGGTAAGGGTGGATAAGCTACCAGTTCACTCCAGAGATATCCCTGGTGGCAGGTGGGTTCTACCATGGA
AoHa    (641) TAGCCTACAGGGGCAAGGTTGGAATCAGCTACCAGTTTACTCCAGAAATATCTTTGGTGGTAGGTGGGTTCTACCACGGA
Apl     (641) TAGCTTACAAAGGTAAGGTTGGTGTTAGCTACCAGTTGACACCTGAAATATCATTGATTGCTGGTGGTTTTTACCATGGC
ApHGE1  (638) TGGCTTATAGAGGAAAGGTAGGCGTAAGTTACAAGCTTACTCCTGAAATATCTTTAATAGCTGGAGGTTTTTACCACGGA
ApHZ    (638) TGGCTTATAGAGGAAAGGTAGGCGTAAGTTACAAGCTTACTCCTGAAATATCTTTAATAGCTGGAGGTTTTTACCACGGA
                721                                                                           800
AmStM   (718) CTATTTGATGAGTCTTACAAGGACATTCCCGCACACAACAGTGTAAAGTTCTCTGGAGAAGCAAAAGCCTCAGTCAAAGC
AcIs    (721) CTTTTCGACGAATCTTATAAGGACATCCCGGCACACAATAGCGTGAATTTTCCCGGAGAAGCGAAAGCCTCAATCAAGGC
AoHa    (721) CTCTTTGACGAGTCTTACAAGGACATACCCGCACATAACAGTGTAAAGTTCCCCGGAGAAGCAAAAGCCTCAGTCAAGGC
Apl     (721) CTATTCGATGAAAAGTTCGAAAACATTCCTGCAAACAATAGGGTAAATTTCGCTGGTGAAGCTTTGGCAACAGTGAAGGC
ApHGE1  (718) ATTTTCGATGAACAGTATGCAGGTATTCCCGCTAGTAACCGTGTAAACATAGCAGGTGGTGCTGCAGCCAAGGTAAAGGC
ApHZ    (718) ATTTTCGATGAACAGTATGCAGGTATTCCCGCTAGTAACCGTGTAAACATAGCAGGTGGTGCTGCAGCCAAGGTAAAGGC
                801                                                   852 rc of AB1693R
AmStM   (798) GCATATTGCTGACTACGGCTTTAACCTTGGAGCAAGATTCCTGTTCAGCTAA
AcIs    (801) GCACGTTGCTGATTACGGCTTTAACCTCGGAGCAAGATTCCTGTTCAGCTAA
AoHa    (801) ACATATTGCTGATTACGGTTTTAACCTTGGAGCAAGATTCCTGTTCAGCTAA
Apl     (801) TAACATTGCAAGTTATGGATTCAATCTGGGAGCAAGATTCGCATTTAATTAA
ApHGE1  (798) AAATATAGCTAGTTACGGCTTCAACATAGGAGCAAGATTTGCTTTCAATTAA
ApHZ    (798) AAATATAGCTAGTTACGGCTTCAACATAGGAGCAAGATTTGCTTTCAATTAA
```

Figure S3: Alignment of *msp4* sequences from several species of *Anaplasma*. The sequences were taken from genome sequences for each species. Abbreviations and genome accession numbers are as follows: AmStM = *A. marginale* St. Maries strain (CP000030); AcIs = *A. centrale* Israel strain (CP001759); AoHa = *A. ovis* Haibei strain (CP015994); Apl = *A. platys* S3 strain (CP046391); ApHGE1 = *A. phagocytophilum* HGE1 strain (APHH00000000); ApHZ = *A. phagocytophilum* HZ strain (CP000235). The underlined regions show the primers used in the study. The reverse complement (rc) of the reverse primer is underlined. The numbers above each row indicate the alignment position number. The numbers at the start of each row in parentheses indicate the base number of the first base on that row for that sequence.

ankA

```
                2551                                                                              2635
AmStM   (2254)  TGCGCCCGTGGTGCCTCTGCTCGCCAGCGCAATGCTCAGGGCCATACTCCACTGCACATGGCAGCTGCGTACAGGAGTATACCCG
Apl     (2386)  ATGAGACACGGCGCTCGTCCAGAAAATGCAAATGTCGAAGGATATACTGCAATGCATATAGCAGCTTC-GTCGGGCAACCTGGAA
ApHZ    (2197)  TTGGATAAAGGGGCTCCAGCAACGCAGAGAGATGCTAGGGGTAGGACGGCTTTACATATAGCAGCTGC-TAATGGTGACG-GTAA
ApHGE1  (2197)  TTGGATAAAGGGGCTCCAGCAACGCAGAGAGATGCTTATGGTAAGACGGCTTTACATATAGCAGCTGC-TAATGGTGACG-GTAA
                2636                                LA6-F                                         2720
AmStM   (2339)  CATTTGAGAGCATATTGAGGAGTAACAAGCTCAGTGCACTGGAGCGCTCCAGCGTTGAT---GGGTCTTCGGCGCTACACGTTGC
Apl     (2470)  ATGATAGGCG-TGATAGTGAAGCATCATCCAGA-AGCATTAATGCACAGGACAGCGTCTACGCACAGCACCATATTCCATGAGGC
ApHZ    (2280)  GCTATATAGGATGATTGCGAAAAAATGCCCAGATAGCTGTCAACCACTCTGTT-CTGATATGGGAGATACAGCGTTACATGAGGC
ApHGE1  (2280)  GCTATATAAGTTAATTGCGAAAAAATGCCCAGATAGCTGTCAAGCACTCCTTT-CTCATATGGGAGATACAGCGTTACATGAGGC
                2721                                                                              2805
AmStM   (2421)  CATGAAGAGCGGCGTGGGT---GAAAGTGACATTATGAGAATGCTGAAGAGCTGCCAGCAAATTTTAGCTCCAGTTGAGCTT---
Apl     (2553)  TATTCGTTCAACGCTGGTCAATGATAAGCAAGTACTGAGGATGTTGCAAATAGCAGAGAAGGGCCTGAGTG---CTGAGCAGTT-
ApHZ    (2364)  TTTATATTCTGATAATGTTACAGAAAAATGCTTTTTAAAGATGCTT-AAAGAGTCTCGAAAG-CATTTGTCAAACTCATCTTTTT
ApHGE1  (2364)  TTTATATTCTGATAAGGTTACAGAAAAATGCTTTTTAAAGATGCTT-AAAGAGTCTCGAAAG-CATTTGTCAAACTCATCTTT--
                2806                                                                              2890
AmStM   (2500)  --GCACAGATGTTGAGCA---CACATGATTCAAGCGGCAACACCCTGATGCATGTTGCTGCGAAGCTGGATTTCAA-AGATGTCG
Apl     (2634)  --CAAGAAC--CTAATCAATGCCCAAGACATAAAGGGTAACACCTTAATGCACATGGCAGCAAGTAAGGGAAGCCG-AAAACTGG
ApHZ    (2447)  TCGGAGACTTGCTTAATACTCCTCAAGAAGCAAATGGTGACACGTTACTGCATCTGGCTGCATCGCGTGGTTTCGGTAAAGCATG
ApHGE1  (2445)  -CGGAGACTTGCTTAATACTCCTCAAGAAGCAAATGGTGACACGTTACTGCATCTGGCTGCATCGCGTGGTTTCGGTAAAGCATG
                2891                                                                              2975
AmStM   (2579)  TGGCGTTTGGCCTGAACAATGGCGCACAGATGTCTATTAAAAACGCAGAGGGTAAACTTGCTCACGAGCTAGCTCCACCATCAGG
Apl     (2714)  TTAATTACCTGATGCAGCACGGTGCTAGTCTGTCAATGGCGAATATCAAAGGAAATACAGCAACGAATCTGGTGAAAGATGCAAG
ApHZ    (2532)  TAAAATAC-TACTAAAGGCTGGGGCGTCAGTATCAGTCGTGAATGTAGAGGGAAAAACACCGGTAGATGTTGCG---GATCCATC
ApHGE1  (2529)  TAAAATAC-TACTAAAGTCTGGGGCGTCAGTATCAGTCGTGAATGTAGAGGGAAAAACACCGGTAGATGTTGCG---GATCCATC
                2976                                                                              3060
AmStM   (2664)  CCACATAGCGGGCGGCAAGTTAGCTAGCAAGATGGCCACACAGGTCAACAGCGAGGGAGGAGCTCGTAGCTACAGCACAGTTACA
Apl     (2799)  GTTCATAAGTAAGGGGTTGTTTAAAAAACAGT------CTCTGTGGG-AAGAAATGAAATCACGAGAA--------ATCAGGCCA
ApHZ    (2613)  ATTGAAAACTCGTCCGTGGTTTTTGGAAAGT------CCGTTGTCA-CAATGATGGCTGAACGTGTT--------CAAGTTCCT
ApHGE1  (2610)  ATTGAAAACTCGTCCGTGGTTTTTGGAAAGT------CCGTTGTCA-CAATGATGGCTGAACGTGTT--------CAAGTTCCT
                                                          rc of LA1-R
```

Figure S4: Alignment of *ankA* sequences from a few *Anaplasma* species showing the region that was amplified in this study. The sequences were taken from genome sequences for each species. Abbreviations and genome accession numbers are as follows: AmStM = *A. marginale* St. Maries strain (CP000030); Apl = *A. platys* S3 strain (CP046391); ApHZ = *A. phagocytophilum* HZ strain (CP000235); ApHGE1 = *A. phagocytophilum* HGE1 strain (APHH00000000). The underlined regions show the primers used in the study. The reverse complement (rc) of the reverse primer is underlined. The numbers above each row indicate the alignment position number. The numbers at the start of each row in parentheses indicate the base number of the first base on that row for that sequence.
